# Chiral‐Induced Spin Selectivity Effect in a 1 nm Thin 1,1′‐Binaphthyl‐2,2′‐diyl Hydrogenphosphate Self‐Assembled Monolayer on Nickel Oxide

**DOI:** 10.1002/advs.76252

**Published:** 2026-06-23

**Authors:** Abin Nas Nalakath, Christian Pfeiffer, Anu Gupta, Franziska Schölzel, Michael Zharnikov, Georgeta Salvan, Ron Naaman, Marc Tornow, Peer Kirsch

**Affiliations:** ^1^ Organic Electronics Technical University of Darmstadt Darmstadt Germany; ^2^ Molecular Electronics Technical University of Munich Garching Germany; ^3^ Department of Chemical and Biological Physics Weizmann Institute of Science Rehovot Israel; ^4^ Institute of Physics Chemnitz University of Technology Chemnitz Germany; ^5^ Research Center for Materials, Architectures and Integration of Nanomembranes Chemnitz University of Technology Chemnitz Germany; ^6^ Applied Physical Chemistry Heidelberg University Heidelberg Germany; ^7^ Merck Electronics KGaA Darmstadt Germany; ^8^ Freiburg Materials Research Center (FMF) University of Freiburg Freiburg Germany

**Keywords:** chiral phosphoric acid, chiral‐induced spin selectivity, circular dichroism, magnetic conductive atomic force microscopy, self‐assembled monolayers

## Abstract

The chiral(ity)‐induced spin selectivity (CISS) effect describes the observed correlation between the spin of an electron transferred through a molecule and that molecule's chirality. Primary CISS systems are based on self‐assembled monolayers (SAMs) of multiple nanometer‐long biomolecules exhibiting thiol‐anchored helical chirality. For transforming the concept to real application, more robust molecule‐substrate systems are required. Phosphonic and phosphoric acid SAMs coupled to metal oxides can provide the necessary robustness. In this work, we report on studies employing the aromatic, axially chiral organophosphoric acid derivative 1,1′‐binaphthyl‐2,2′diyl hydrogenphosphate (BNP). Grown as a roughly 1 nm thin SAM on top of NiO_x_/Ni substrates, the system exhibits a high CISS‐magnetoresistance (CISS‐MR) of 50%–80% when measured using magnetic‐conductive atomic force microscopy. For biases above 0.5 V, the magnetoresistance curves could be fitted to a minimal Fowler‐Nordheim (FN) tunneling model. From this model, we determined that, depending on the molecules’ handedness, electrons of a certain spin direction face an effective tunneling barrier, which is either 80% higher or 40% lower compared to the barrier for electrons of opposite spin direction. Due to their small size, compatibility with oxide materials, and commercial availability, these molecules are excellent candidates for the realization of novel organic spintronic devices.

## Graphical abstract

A self‐assembled monolayer (≈0.9 nm) of an axially chiral binaphthol phosphoric acid derivative on a ferromagnetic Ni/NiO_x_ substrate shows chiral‐induced spin selectivity (CISS) magnetoresistance of 50%–80%. This thermally and chemically robust functional stack shows the way towards practical spintronic devices based on the CISS effect.

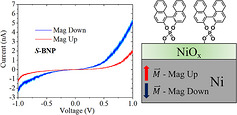

## 1 Introduction

**FIGURE 1:**
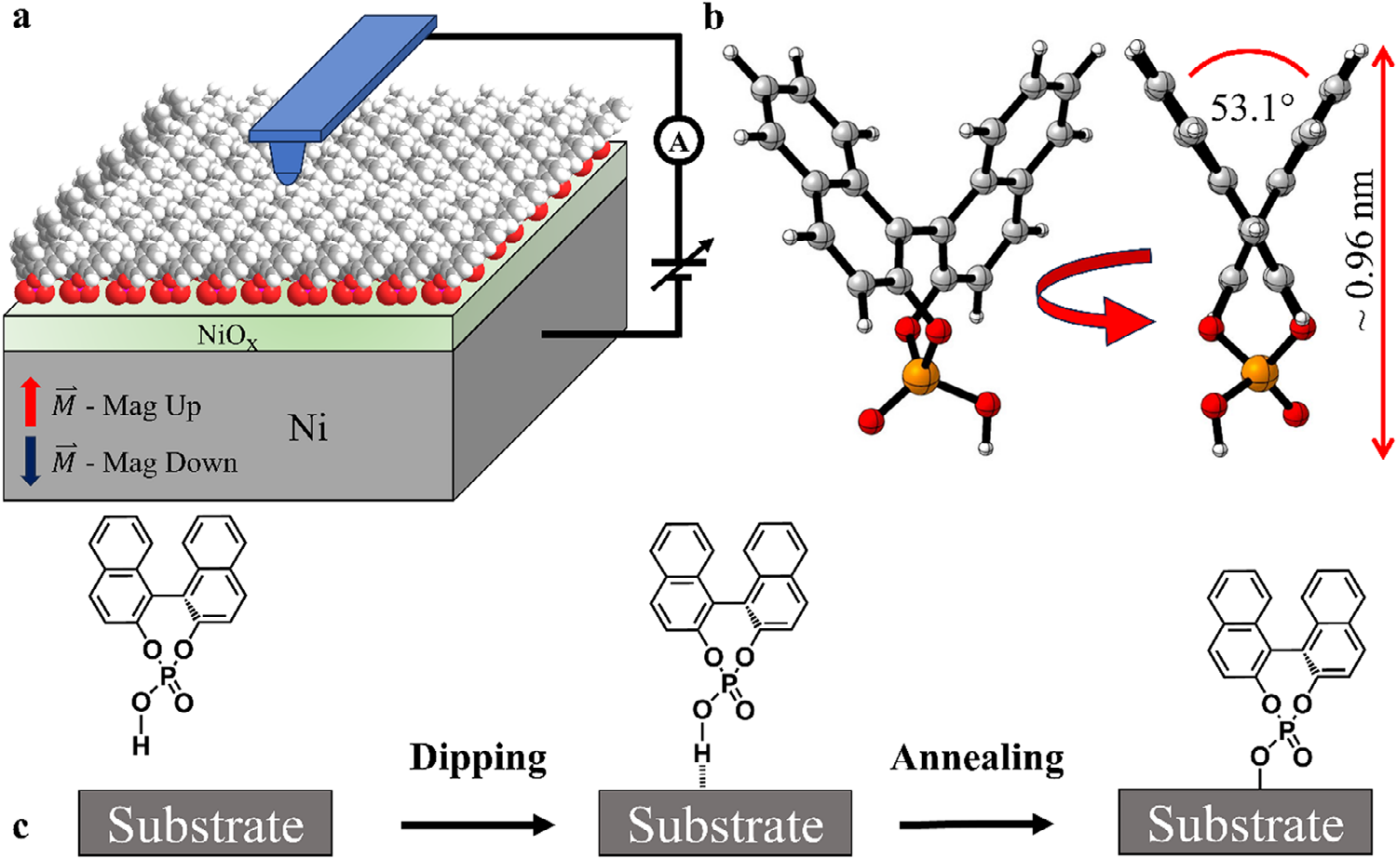
(a) Schematic illustration of mc‐AFM setup for electrical, magnetic field‐dependent CISS‐MR measurements. Si, Ti, and Au layers below Ni are omitted. Dimensions are not drawn to scale. Depiction of *R‐*BNP within SAM is taken from Chem3D (Revvity, Waltham, USA). (b) Rendering of the *S‐*BNP molecule visualized from two different viewing angles [40]. (c) Illustration of the self‐assembly process of BNP on NiO_x_. This process involves a dipping and an annealing step to facilitate a covalent bond formation between the phosphoric acid group and the native metal oxide substrate (only one of several possible binding modes is shown).

Since the discovery of the chiral(ity)‐induced spin selectivity (CISS) in 1999 [1], the effect has been observed in a wide variety of chiral materials and compounds [2, 3]. A standard configuration for detecting CISS consists of a ferromagnetic Ni substrate (often capped by a thin Au layer) and few‐nanometer‐long thiolated biomolecules, such as double‐stranded DNA or polypeptides, deposited as self‐assembled monolayers (SAMs) [4, 5, 6, 7]. It has been reported that the CISS response in α‐helical oligopeptides and in double helix DNA increases linearly with the length of the helix [8]. Consequently, several theoretical approaches utilize helical chirality as a basis for modelling CISS [9, 10, 11, 12]. However, while indeed many studies have focused on few‐nanometer‐long α‐helices, CISS has been observed in all types of chirality [2], including one‐atom thick layers with 2D chirality [13]. Besides continuous efforts towards a better understanding of the fundamental mechanisms of the CISS effect [2, 14], its potential role in forthcoming spintronic and quantum applications has moved to the foreground recently [15, 16, 17]. Electronic devices based on the manipulation of the spin‐polarized electrical currents (spintronics) have been in use since the early 2000s [18], with main applications in the areas of memory and information processing [19, 20, 21]. While a memory structure based on a chiral layer has been proposed previously [22], device architectures competitive to the spintronics state‐of‐the‐art, including magnetic tunnel junctions (MTJs), giant magnetoresistance (GMR) junctions, and related, mature industrial‐scale technologies, are yet to be demonstrated. This is partially related to the fact that most CISS‐magnetoresistance (CISS‐MR) setups involve a noble metal directly deposited on the ferromagnetic substrate, resulting in, for example, Au/Ni [23] or Au/Co/Au [5] stacks. These stacks, however, are not compatible with many semiconductor fabrication processes, as they contain Au, which is not CMOS‐compatible [24, 25]. In addition, the employed helical biomolecule thiols lack sufficient robustness against thermal or oxidative stress. In this context, the ability to utilize short non‐helical and even non‐bioorganic materials, which would demonstrate a robust CISS signature in transport, and their combination with a substrate stack that avoids the use of Au, appears to be particularly relevant [16, 26]. Hence, in this work, we set out to design and demonstrate such a system, which is both chemically robust and compatible with most microelectronics fabrication processes. It relies on the use of phosphoric molecule‐based SAMs, chemically related to organophosphonate SAMs, for which we have shown that binding them to semiconductor and metal oxides is a robust and reliable pathway for making them an integral part of various functional electronic devices [27, 28, 29, 30]. The covalent binding mechanism between the acidic OH moiety of the molecules’ anchoring group and surface oxides, as well as the charge transport through phosphonic acid molecules, and their ability to serve as robust passivation layers against oxidation, have been extensively studied [31, 32, 33, 34, 35]. Consequently, for the chiral SAM, we were looking for a simple compound that shows high chirality, is readily available in both enantiomers, and can bind covalently to metal oxides. Among several potential candidates, suitable compounds in this context are cyclic 1,1'′bi‐2‐naphthol (binol) derivatives, which are known in liquid crystal technology for their extraordinary ability to induce chirality into nematic phases [36, 37]. Therefore, we selected commercially available binol phosphoric acid (BNP) [38] as the chiral component on Ni substrates. BNP ‐ as illustrated in Figure 1b and less than 1 nm short ‐ manifests the axial chirality of the binol moiety. The two naphthalene arms are rotated against each other by 53.1° [39]. An overview of the deposition process, which involves immersion in the solution, annealing, and subsequent covalent bonding with the metallic oxide surface, is shown in Figure 1c. A detailed description of the SAM deposition procedure is provided in the Supporting Information. The architecture of the entire system is illustrated in Figure 1a along with the used magnetic‐conductive atomic force microscopy (mc‐AFM) measurement setup.

We here provide proof of concept for a novel device architecture utilizing the CISS effect, which introduces both a substrate material (metal/metal oxide), which is compatible with most common microelectronics fabrication processes, and an extremely thin synthetic, chiral organic compound monolayer. Not only is the chirality of these molecules preserved upon self‐assembly, but mc‐AFM measurements indicate strong CISS responses with a CISS‐MR > 50%. This finding is remarkable since the substrate material has much lower spin‐orbit coupling (SOC) than commonly used Au, which therefore points to the apparently limited influence of the substrate SOC on the extent of the CISS effect. We anticipate our results to provide a viable platform for future CISS‐based spintronic applications.

## 2 Results

### 2.1 Surface Analysis

**FIGURE 2:**
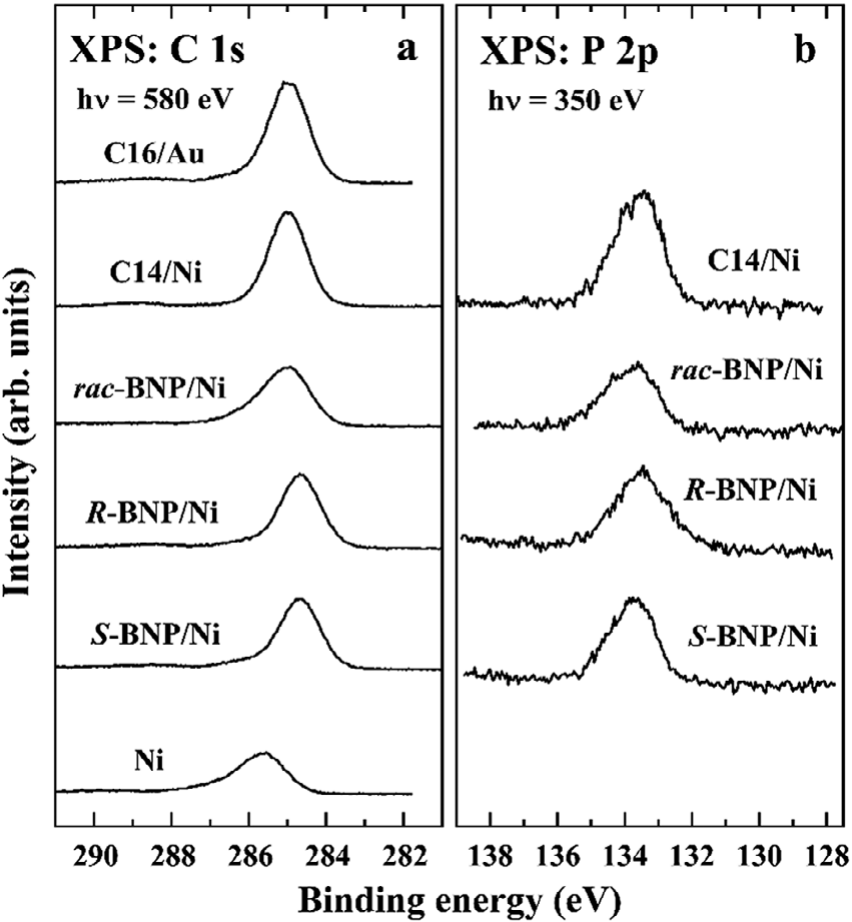
C 1s (a) and P 2p (b) XPS spectra of *S‐* and *R‐*BNP on Ni, individually and as a racemate (*rac*), along with the reference spectra of C14/Ni, C16/Au, and bare Ni.

To verify the presence of BNP on NiO_x_, we conducted X‐ray photoelectron spectroscopy (XPS) measurements. As expected, according to the Ni 3p spectra (see Figure S1), the surface of the Ni substrates is oxidized. The thickness of the oxide layer, estimated from the relative weight of the oxide contribution in the entire Ni 3p signal, is 1.3 nm (see the Supporting Information, section 2). The oxidation state of Ni is less than in stoichiometric NiO, as emphasized by the lower binding energy (BE) of the Ni 3p_3/2_ signal (67.27 eV vs. 69.00 eV for NiO [41]). The C 1s and P 2p spectra of *S‐* and *R‐*BNP on Ni, individually and as the racemate, are presented in Figure 2a,b, respectively. These spectra are referenced against the spectra of *n‐*tetradecyl phosphonic acid (C14) on Ni and *n‐*hexadecanethiolate (C16) SAM on Au(111) (C 1s only). The latter was prepared by a standard procedure [42], features well‐defined properties [43], and serves as an established reference for SAM studies [44]. All carbon spectra in Figure 2a exhibit a single peak at either ∼284.7 eV or 284.96 eV corresponding to binol and alkyl backbones, respectively, and differing from the signal of carbon contamination for the blank Ni, recorded at 285.7 eV (we assume that most of this contamination was wiped off upon the SAM formation). Whereas the peaks for C14/Ni look symmetric, those of the BNP films are accompanied by slight shoulders (residual contamination and, probably, the carbon atoms bound to the anchoring group), especially pronounced in the case of *rac‐*BNP. Significantly, the intensities of the C 1s signal for the films on Ni are lower than that of the C16 thiol‐SAM, which suggests that all former films are monolayers. The numerical evaluation of the C 1s data, using the standard expression for the self‐attenuation of the photoemission signal [45], literature values for the attenuation lengths in SAM‐like films [46], and the thickness of C16/Au (1.89 nm) [43] as a reference, gives the thicknesses of the C14 and BNP films (hydrocarbon matrix only) of ∼1.35 nm and ∼0.92 nm, respectively. These values correlate well with the lengths of the molecular backbones, which are ∼1.8 nm (calculated based on length per methylene group [47]) and 0.96 nm, respectively. The P 2p spectra of all films on Ni in Figure 2b exhibit a single P 2p_3/2,1/2_ doublet at a binding energy of ∼133.5 eV (P 2p_3/2_), corresponding to the anchoring phosphoric acid groups.

However, these data do not allow us to derive information about the exact binding mode of these groups to the substrate [48], which can generally involve a variety of different motifs, from monodentate to tridentate [33, 49]. Yet, we can estimate the packing density of the BNP monolayers based on these data, assuming that the packing density of C14/Ni is similar to that of C16/Au (4.63 · 10^14^ molecules·cm^−2^) [43]. Calculating the intensities of the P 2p signals and taking into account the attenuation by the binol/alkyl matrix, we got a packing density of $∼1.9\cdot 10^{14}$ molecules·cm^−2^ for the *R‐*BNP and *S‐*BNP monolayers and ∼ 1.65 · 10^14^ molecules·cm^−2^ for the *rac‐*BNP film, which is in good agreement with the layer structure in *R‐* and *S‐*BNP crystals featuring 1.817 · 10^14^ molecules · cm^−2^ (see also Supporting Information) [39]. Thus, the packing density of the racemate film is ∼ 20% lower than that of the enantiomer monolayers. This could be due to the triclinic space group crystallization known to take place in *rac*‐BNP [50], which stands in contrast to the layered crystalline structures that occur in enantiopure BNP [39].

Complementary information about the properties of the BNP films is provided by near‐edge X‐ray absorption fine structure (NEXAFS) data (see Figures S2 and S3), using the established approach [51]. Here, the BNP samples show the characteristic absorption features of naphthalene, which is the major building block of binol. The small but distinct dichroism in the spectra of the enantiomer SAMs suggests a certain degree of orientational order and upright molecular orientation. In contrast, the lack of any dichroism in the *rac*‐BNP spectra indicates a more disordered monolayer compared to the enantiomer samples.

**FIGURE 3:**
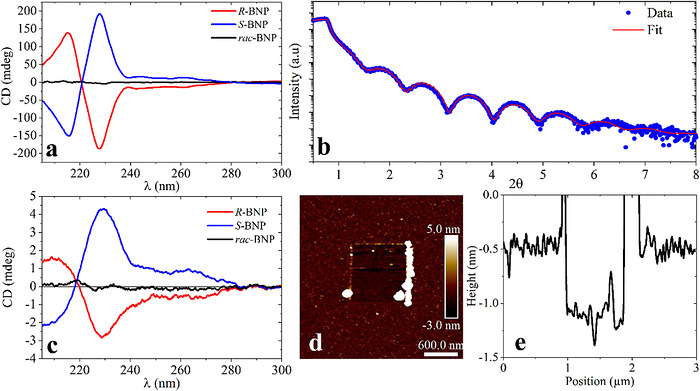
Characterization of the BNP molecules and the respective monolayers on NiO_x_: (a) CD spectra of *R*‐BNP (red), *S*‐BNP (blue), and *rac*‐BNP (black) in ethanol (0.01 mM solution). (b) XRR results for *rac*‐BNP grown as a SAM on NiO_x_/Ni substrate. Measured reflected intensity is drawn in blue circular markers over the angle of incidence 2θ. The red line indicates the fit. (c) CD spectra of *R*‐BNP (red), *S*‐BNP (blue), and *rac*‐BNP (black) monolayers grown on NiO_x_/Ni/sapphire. (d) 3 × 3 µm^2^ AFM tapping recording of a scratched sample of an *S*‐BNP monolayer. (e) Mean height profile along the horizontal direction over the scratched area in (d).

The surface roughness analysis of bare and BNP‐coated samples, as determined using AFM, is presented in Figure S4. Furthermore, AFM‐based scratching experiments were conducted, in which the applied force of an AFM tip was calibrated to mechanically remove the soft organic monolayer in contact mode. Figure 3d depicts a 3 × 3 µm^2^ tapping mode image of a scratched *S‐*BNP‐coated sample, indicating successful removal of the organic layer in the central square. Height variations along the horizontal axis of the image are averaged over the scratched area and shown in Figure 3e. Calculated as the height difference between the scratched and unscratched parts in the profiles, the *S*‐BNP layer is determined to be 0.6 nm thin. Additional height analysis for samples with *R*‐BNP and *rac*‐BNP is shown in Figure S5. It yields monolayer thickness values of 0.5 nm, 0.6 nm, and 0.8 nm for *rac*‐BNP, *S*‐BNP, and *R*‐BNP, respectively. Grazing incidence X‐ray reflectivity (XRR) measurements were performed at different positions to evaluate the structural uniformity of the films. In Figure 3b, a representative XRR recording is shown with its corresponding simulated fits. The simulated profile closely matches the experimental Kiessig fringes, confirming the accuracy of our fitting procedure. The fit is not only able to reproduce the thicknesses specified for the substrate wafer but also determines a NiO thickness of 1.33 nm and a SAM thickness of 0.98 nm, which is in good agreement with XPS and AFM data (see Tables S1–S3 for comparison).

### 2.2 Circular Dichroism

The CD spectra for the BNP molecules in ethanol solution are presented in Figure 3a. They show mirror‐image Cotton effects for the enantiomers, while the racemic mixture is optically inactive. The degree to which the chirality is expressed optically can be quantified by the dissymmetry factor *g* [52, 53], defined as *g*  =  Δε/ε, where Δε is the difference in the molar absorption coefficient for left and right circularly polarized light and ε is the molar absorption coefficient of the molecule [54]. Recent work on the twisted‐acene systems shows that the sign and magnitude of the CD peaks (associated with *g*) qualitatively track the sign and magnitude of the CISS‐derived spin polarization, suggesting that the factor *g* captures the chiral electronic response which underlies spin selectivity [55]. Notably, while the parameters ε and Δε scale with the number of naphthyl chromophores, the dissymmetry factor offers a normalized measurement of chirality. For the *R‐*BNP enantiomer, there is a positive band at 215.5 ± 0.1 nm (θ ≈ 150 mdeg, *g* ≈ 1.6 · 10^−3^), followed by a negative band at 227.5 ± 0.1 nm (θ ≈ −191 mdeg, *g* ≈ −2.5 · 10^−3^). [Correction added on 13 August 2026, after first online publication: in the above sentence, the numbers −150 and −1.6 were changed to 150 and 1.6, while 0.1 and 2.5 were changed to −0.1 and −2.5.] *S‐*BNP exhibits the practically exact mirror‐image response. The close alignment of the absorption maxima and the mirror‐image CD signals support the conclusion that the observed optical activity results from exciton coupling between the naphthyl transition dipoles. The magnitudes of *g* ($∼10^{-3}$) align with those reported for similar binaphthyl frameworks [56, 57, 58], highlighting the strong intrinsic chirality of BNP. This behavior is consistent with findings by Amsallem et al., which demonstrate that thiolated binaphthalene and ternaphthalene exhibit different ε and Δε values, yet have similar *g* factors that correlate with comparable spin polarization efficiencies [56]. Additionally, the relationship between BNP's absorption bands and its mirror‐image CD signals matches the behavior observed in other binaphthyl derivatives during light‐driven desorption experiments [59].

Importantly, we also recorded CD spectra of *R‐*BNP, *S‐*BNP, and *rac‐*BNP in SAMs on NiO_x_/Ni/sapphire. The CD measurements show the chirality of the SAM on the surface and confirm that the BNP SAM preserves its chiral nature after adsorption. As shown in Figure 3c, the overall spectral shape is preserved when probed in monolayer form, consistent with the solution CD spectra (Figure 3a), and the enantiomers continue to exhibit roughly mirror‐image CD bands in the 220–260 nm region.

The CD spectra of the thin films showed maximum ellipticities of ± 3 − 4 mdeg, that were weaker than those observed in solution (Figure 3c). Additionally, the spectral bands of the thin films were significantly broader than those of the corresponding CD spectra in solution. The *g*‐factors of the thin films were determined to be 5.0 · 10^−5^ for *S‐*BNP and 5.2 · 10^−5^ for *R‐*BNP. The reduction in the g‐factor for the thin films can be attributed to the experimental configuration, which includes both the substrate and the SAM. The nickel substrate shows strong absorption and reflection in the UV region [60]. As in the calculation of the g‐factors of the thin films, reflection is not taken into account, the genuine g‐factors of the thin films might in fact be larger than the experimentally estimated value. Both enantiomeric thin films exhibited similar trends in their solution CD spectra. In contrast, the racemic films showed no significantly detectable CD signal, indicating the absence of overall chirality in the system, consistent with the solution‐state CD results.

### 2.3 CISS‐Magnetoresistance

**FIGURE 4:**
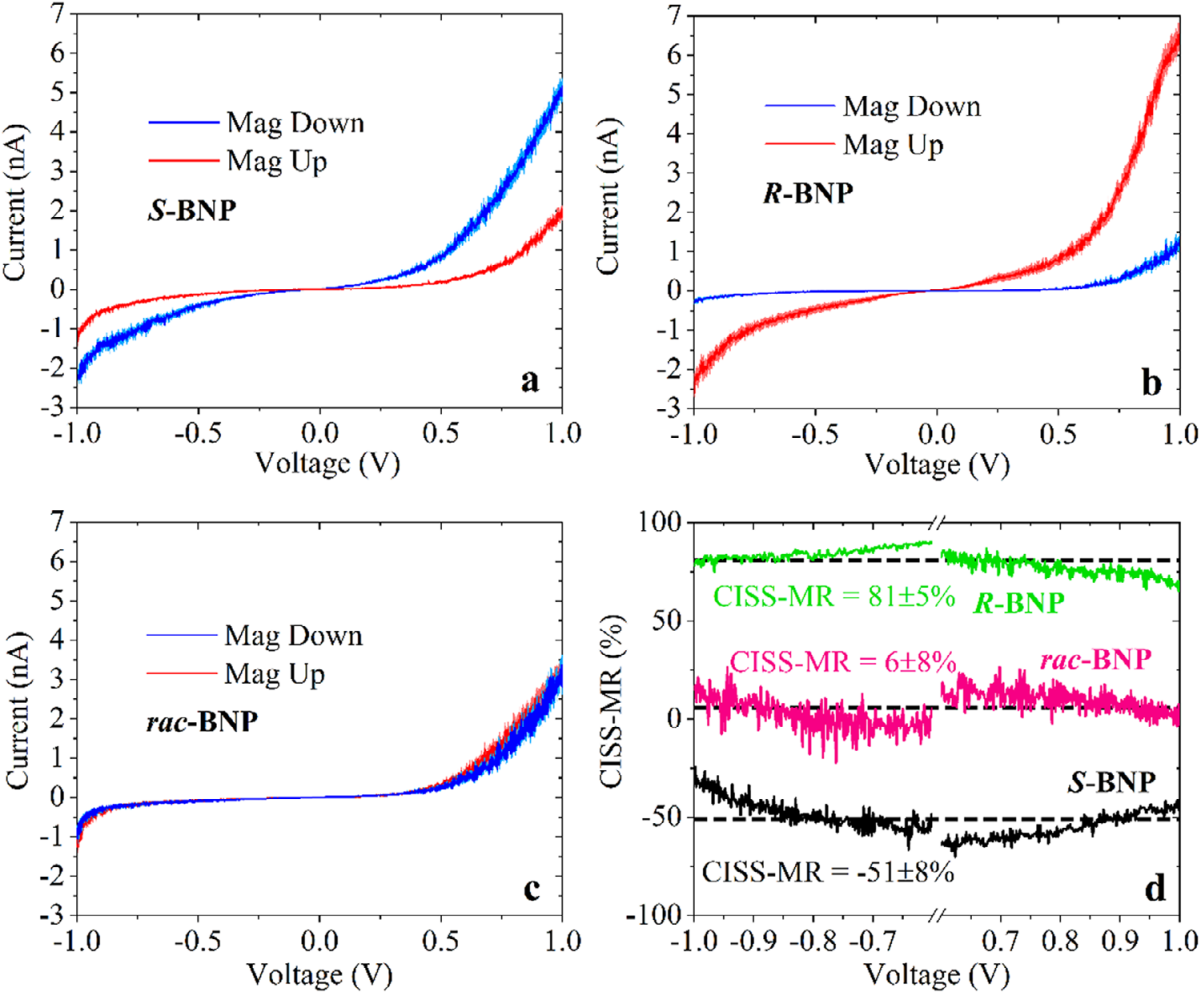
Magnetic field‐dependent mc‐AFM measurements of the *S‐*BNP (a), *R‐*BNP (b), and *rac‐*BNP (c) SAMs. Blue (red) lines represent average IV curves when the magnetic field (0.5 T) is pointed downwards (upwards), perpendicular to the sample plane. Solid lines indicate the mean curves. Shaded, lighter areas indicate the standard error of the mean. (d) Determined CISS‐MR for *R‐*BNP (green), *S‐*BNP (black), and *rac*‐BNP (magenta) for voltages between −1 and −0.6 V, and between +0.6 and +1 V.

Figure 4a,b show the mc‐AFM results for the *S‐*BNP and *R‐*BNP SAMs, respectively. As is visible, the current magnitude depends on the magnetization of the underlying Ni layer. While the current passing through downward magnetized Ni seems to be favored by *S‐*BNP (Figure 4a), *R*‐BNP (Figure 4b) exhibits the opposite behavior. For samples coated with *rac‐*BNP, the measured magnetoresistance appears to be statistically insignificant, with overlapping error bars (Figure 4c). The individual IV‐curves measured for *S‐*BNP, *R‐*BNP, and *rac‐*BNP are provided in Figures S8–S10, respectively. It is noteworthy that, while the results in Figure 4 are based on statistics of multiple positions, the measurements on each individual position also exhibit the expected CISS response. The measurements taken for *rac*‐BNP (Figure S10) do show small CISS‐MR effects on individual positions but appear insignificant when averaged. We attribute this to a local excess of one enantiomer for individual mc‐AFM junctions. As is visible in Figures S8–S10, several IV measurements reach the measurement system's maximum limit of 10 nA. This can lead to an underestimation of the mean current for applied bias above 0.5 V. Using the currents measured when the external magnetic field is pointed upward, here denoted as *I*_↑_ and downward *I*_↓_, respectively, CISS‐MR in per cent can be calculated according to

(1) $$CISS-MR=\frac{I_{↑}-I↓}{I_{↑}+I_{↓}}\cdot 100$$

The resulting CISS‐MR is plotted in Figure 4d for *R‐*BNP (green), *S‐*BNP (black), and *rac*‐BNP (magenta) for absolute voltages between 0.6 V and 1 V. This yields a CISS‐MR for *R‐*BNP of ≈ 81 ± 5%, for *S*‐BNP of ≈ −51 ± 8%, and for *rac*‐BNP of ≈ −6 ± 8%. These values represent the average and standard deviation of the respective data points shown in Figure 4d. The three results for the CISS‐MR, as calculated over the entire voltage range, are provided in Figure S11.

## 3 Discussion

The measured CISS‐MR is roughly in the range of values for thiol‐functionalized binaphthyl monolayers [56], underscoring the robustness of axial chirality in inducing spin selectivity. If we consider the thickness of the BNP SAM alone, and the previous observation that CISS increases with the length of a helical structure [8, 61, 62], the achieved MR is remarkably high. Notably, the measured magnitude falls within the range of comparable values for roughly 12 nm long dsDNA and exceeds that for oligopeptides of > 2.5 nm length [8].

A unique characteristic of the BNP system is the intrinsically defined orientation of the individual molecules on the surface: BNP has basically no conformational degrees of freedom, and the binding to the substrate can occur only through the phosphate moiety. Therefore, it can be expected that the chiral surface looks actually very similar to the layers observed in the crystal structures of *R‐* and *S‐*BNP (see Figure S6) [39]. BNP has an approximate *C*_2_ symmetry with a continuous symmetry measure (CSM) *S*(*C*_2_) of 0.9309 and a continuous chirality measure (CCM) of 8.3447 [63]. On binding to the substrate, the *C*_2_ axis of BNP is ideally oriented perpendicularly to the substrate plane.

As noted above, the molecules are grown onto the native oxide of Ni, which is, in principle, expected to be NiO with at least partial hydroxyl termination [64, 65]. Therefore, the spin‐polarized current injected from the ferromagnet must pass an oxide barrier. Bulk stoichiometric NiO has a wide band gap (∼3.6 – 4.0 eV) [66, 67, 68] and acts as a Mott insulator [69]. If this applies equally to the native thin film oxide, the electrons would first have to pass through a potential barrier before entering the chiral layer, resulting in a TMR [70, 71] effect instead of a GMR [72, 73] effect. XPS data indicate that this native oxide exists in a slightly sub‐stoichiometric oxidation state. It has been previously shown that in NiO_x_, a lower oxidation state can result in higher resistance [74, 75]. However, especially in ultrathin films of ordered NiO, smaller effective band gaps and stronger coupling to the metal have been reported [76, 77]. Further, a narrower effective gap can also be correlated with a lower level of ordering and non‐ideal stoichiometry [78], which is expected for the natively grown oxide film, such as on the present substrates. We therefore conclude that the thin layer between the ferromagnetic metal and the chiral layer may be significantly less insulating than bulk NiO. This would mean that the measured difference in resistance is closer to a GMR than to a TMR.

Nickel oxide is known to have antiferromagnetic characteristics at room temperature [79, 80, 81]. Generally, this could lead to a depolarization of the current injected into the SAM due to the short spin decay lengths in antiferromagnets [82]. However, assuming that spin‐polarized current is governing the CISS effect, the measured MR does not indicate any strong antiferromagnetic properties dominating the charge transport through the NiO_x_. This could be due to the strong dependence of the Néel‐temperature on the layer thickness. For example, a five‐monolayer‐thick film of epitaxially grown NiO on MgO has a reduced Néel‐temperature of 295 K [83]. Altieri et al. reported a Néel temperature below 40 K for three monolayers of NiO grown on MgO and a comparatively high temperature of 390 K when grown on Ag [84].

As pointed out previously, the measure of *CISS‐MR* (also sometimes referred to as spin polarization), as calculated in Equation (1), does not refer to the spin polarization induced by the chiral molecule alone [85], but rather reflects the differences in resistance of the entire stack based on the direction of the magnetic field. Notably, the CISS effect has also been observed in one‐atom‐thick molecular layers with 2D chirality [13] or in TMR junctions with chiral molecules [86]. In the latter study, the CISS was explained by a spin‐polarized or spin‐selective interface effect. Importantly, in many cases, the IV curves are anti‐symmetric with respect to the bias voltage, that is, *I*(+*V*) is equal to ‐*I*(‐*V*).

The observed non‐anti‐symmetry in our measurements may stem from the different work functions and electronic structures of the materials in our stack: Ni (5.15 eV) [87], NiO (band gap ≈ 4.3 eV [88], work function ≈ 5.2–6.7 eV [65]), and Pt (5.65 eV) [87]. Rikken and Avarvari commented on the role of device asymmetry in CISS‐MR results, pointing out that while the electrode asymmetry appears to be a prerequisite for CISS‐MR, results typically still report antisymmetric IV curves, possibly due to the effect being below the detection limit of common mc‐AFM techniques [89]. In fact, to a certain extent diode‐like IV curves under a magnetic field are generally associated with the electrical magnetochiral anisotropy (eMChA) effect [89, 90], indicating that it may have contributed to the results in Figure 4. However, the results presented here show that the polarity of the current rectification is independent of the applied magnetic field, suggesting that eMChA does not significantly affect the observations above.

In many nanometer‐thin SAMs, coherent tunneling has been reported as the predominant charge transport mechanism [91, 92, 93]. Here, electrons are expected to directly traverse a rectangular barrier (at low bias) in a single step. The characteristic feature of this model is exponential current attenuation with distance according to the relation for the conductance *G*

(2) $$G=G_{C}\cdot \exp\left(-\beta d\right),$$

where *G_C_* is a reference conductance, namely the zero‐length (contact) conductance, *d* is the thickness of the tunnel barrier, and *β* is the junction‐characteristic current decay coefficient [94]. Exemplary, by systematically measuring alkanethiols of varying lengths, a mono‐exponential current attenuation was observed with a decay constant of $\beta \approx 0.5-1\;{Å}^{-1}$ [95].

**FIGURE 5:**
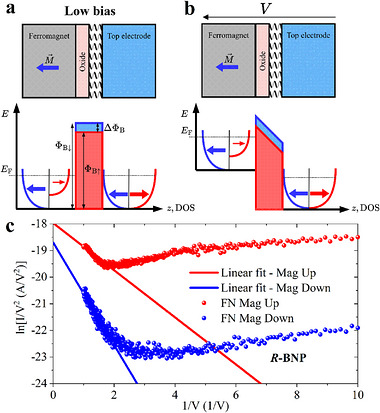
Simplified tunneling models, adapted to CISS: (a) Schematic illustration of phenomenological direct tunneling model at low bias. Case for downwards pointing magnetization and *R*‐chirality is shown. Parabolas indicate the densities of states (DOS) with spin down (blue) and spin up (red) in the ferromagnet and top electrode. Due to zero or low bias, the Fermi levels of the ferromagnet and top electrode are aligned. The tunneling barrier is made up of oxide and SAM. In this reduced model, electrons with spin down face a higher barrier ($\phi_{B↓}$, blue) than electrons with spin up ($\phi_{B↑}$, red). The difference in barrier height is denoted as $\Delta \phi_{B}.$ (b) Schematic illustration of the phenomenological FN tunneling model. The triangular FN tunneling barrier is indicated to be spin‐dependent. Due to applied bias, the Fermi levels are no longer aligned. (c) FN plot for mc‐AFM mean current for *R*‐BNP. Scatter plot indicates data points. Solid lines represent linear fits in the region [1 V^−1^, 2 V^−1^], but are drawn over a larger x‐axis range for better visibility. Blue (red) color refers to currents when the magnetic field is pointing downwards (upwards).

The simplest phenomenological model to assess CISS would also see the chiral surface as a single rectangular tunnel barrier [14]. While the length *d* remains the same for both enantiomers, and in our case, covers the sum of both the oxide barrier and the SAM thickness, the height of the barrier appears different for electrons of opposite spins, which is indicated in Figure 5a. The use of a varying barrier height model to evaluate CISS is well documented in the literature over different material systems and especially in the context of interface effects [90, 96, 97, 98, 99, 100]. In the presented experimental setup, we cannot evaluate *β* as there is no variation of *d*. Further, a fitting for a low‐bias Simmons‐type model [101] would require many critical assumptions, including the exact area of the junction. However, with increasing applied bias, the shape of the tunneling barrier can change, potentially reaching a Fowler‐Nordheim (FN) regime, in which the electron tunnels through a triangularly shaped barrier [94, 102]. This is illustrated in Figure 5b. In a reduced FN model, the current density *J* can then be determined as [103]

(3) $$J=\alpha E^{2}\cdot \exp\left(-\frac{B}{E}\right)$$

with

(4) $$\alpha =\frac{m_{e}}{m^{*}}\frac{q^{3}}{8\pi h\phi_{B}}$$

and

(5) $$B=\frac{8\pi }{3}{\left(2\frac{m^{*}}{h^{2}}\right)}^{1/2}\frac{\phi_{B}^{3/2}}{q}.$$

Here, *E* is the electrical field, *J* the current density, $\phi_{B}$ the effective barrier height, *q* the electron charge, *m_e_* the electron mass, m* the effective electron mass in the barrier materials, and *h* the Planck constant [103].

As can be seen in the FN‐plot in Figure 5c, for an *R*‐BNP‐coated sample, a clear FN‐like regime can be observed. Figure S12 provides the FN plots for both enantiomers and the racemic mixture, showing that, for all samples, the charge transport indeed exhibits an apparent FN regime above 0.5 V (2 V^−1^). The observed linear decrease of $\ln(I/V^{2})$ indicates that FN tunneling may well be the primary transport mechanism in that voltage range. Based on this FN tunneling model, we estimate the ratio of the effective tunneling barriers

(6) $$\frac{\phi_{B↑}}{\phi_{B↓}}={\left(\frac{s_{↑}}{s_{↓}}\right)}^{2/3},$$

where $\phi_{B↑}$ and $\phi_{B↓}$ are the effective barrier heights (rectangular, at zero electric field) calculated for currents *I*_↑_ and *I*_↓_, respectively. The parameters *s*_↑_ and *s*_↓_ represent the slopes of the linear fits to the data within the FN regime, by using the FN relation in Equation (3).

The fitting is based on a minimal model that is described in detail in the Supporting Information. It yields that for *R*‐BNP the ratio of the effective barrier heights is $\frac{\phi_{B↑}}{\phi_{B↓}}=0.6,$whereas for *S*‐BNP it is $\frac{\phi_{B↑}}{\phi_{B↓}}=1.8$. In other words, for *S‐*BNP, electrons with spin up experience an 80% larger tunnel barrier than electrons with spin down. As expected, the ratio is nearly 1 for the racemic mixture. The thereby derived barrier differences are $|\Delta \phi_{B}|\approx 90\;\mathrm{meV}$ for *S*‐BNP and $|\Delta \phi_{B}|\approx 130\;\mathrm{meV}$ for *R*‐BNP, and a mere $|\Delta \phi_{B}|\approx 20\;\mathrm{meV}$ difference for *rac*‐BNP (see Figure 5, Table S4, and the Supporting Information, section 9 for the derivation). We want to emphasize that this determination of absolute barrier differences is based on several simplifying assumptions, such as an effective electron mass of m* ≈ 0.5 *m_e_* (based on reference value in silicon oxide barrier [104]), that the tunnel barrier is made up of both the oxide (∼1.3nm) and the BNP SAM (∼1nm), and that the electric field is uniformly distributed over the entire barrier. Furthermore, the model does not aim at explaining the physical mechanisms of the CISS effect. Notwithstanding that, the above (phenomenological) effective barrier height ratio $\frac{\phi_{B↑}}{\phi_{B↓}}$ remains a reliable figure and we propose it as a quantitative metric, possibly also in future theoretical work.

## 4 Conclusion

To summarize, we demonstrated a spin valve architecture based on a BNP/NiO_x_/Ni stack. The organic monolayer, which comprises the chiral phosphoric acid molecules anchored to the oxide surface, was shown to be roughly 1 nm thin and to have a packing density in the range of 1.6 · 10^14^ −1.9 · 10^14^ cm^−2^ for both enantiomers. The CD response indicates that the chirality is preserved upon self‐assembly. The magnetic‐electrical characterization shows a clearly observable CISS‐based resistance change with a CISS‐MR of ≈50%–80%. We assign this recorded difference in resistance to the (anti)parallel spin polarization in both the magnetic layer and the chiral tunneling barrier. We believe the spin‐selective transport in the latter to be caused by the CISS effect resulting from the axial chirality of BNP. Moreover, we observe a clear fingerprint of FN tunneling as the predominant charge transport mechanism at biases above 0.5 V. The ratio of both effective FN tunneling barriers, each corresponding to either injected spin direction, was derived. This indicates that, phenomenologically, spin‐up electrons face a roughly 80% higher barrier than spin‐down electrons when facing an *S‐*BNP‐based oxide/SAM barrier, whereas the barrier in the same scenario appears to be 40% lower for the *R‐*BNP case. We propose this ratio as a viable measure for the CISS response of a chiral monolayer or interface. It may serve to benchmark different CISS platforms against each other and possibly help further decode the contribution of interface effects on the overall CISS response.

Our presented spin valve architecture demonstrates for the first time that a 1 nm thin SAM of axially chiral organophosphate deposited on a metal oxide, can serve as a basis to a robust CISS device. This result was achieved despite the absence of a helical structure, while at the same time using a substrate with low SOC. Utilizing simple, commercially available binol‐based phosphoric acids, this comprises an important step towards the adoption of CISS‐based devices by the electronics industry. The fabrication of a full solid state CISS device using a chiral SAM is part of an ongoing project and will be subject of a future communication.

## 5 Experimental Section

### 5.1 Sample Preparation

Substrates were provided by SIEGERT WAFER GmbH (Aachen, Germany): Ni (100 nm) / Ti (10 nm) / p^++^‐Si (525 µm) / Ti (10 nm) / Au (100 nm). Before SAM growth, samples were treated either with plasma (80 W, 0.3 mbar, 30 s) or UV‐ozone (4 W, 254 nm). For SAM growth, samples were immersed in 10 mM of BNP dissolved in THF or ethanol for 72 h, then annealed at 80°C for 1 h, rinsed with isopropanol, and again annealed at 80°C for 10 min.

### 5.2 Atomic Force Microscopy

Tapping and scratching images were taken with a Dimension V AFM (Bruker/Veeco, Billerica, Massachusetts, United States) and the diamond‐like‐carbon‐coated tip 190DLC (BudgetSensors, Sofia, Bulgaria).

IV‐curves were taken with a custom‐built mc‐AFM with an electromagnet and a Beetle Ambient AFM. As conductive tips, Pt‐coated DPE‐XSC11 (MikroMasch, Sofia, Bulgaria) were utilized. For magnetic field‐dependent I‐V measurements, a (constant) tip‐surface contact force of 8–10 nN was applied.

### 5.3 X‐ray Photoelectron Spectroscopy

XPS measurements were conducted at the bending magnet HE‐SGM beamline of the synchrotron storage ring BESSY II in Berlin. Experiments were done at room temperature in ultra‐high vacuum (base pressure: ≈1 × 10^−9^ mbar). The binding energy scale is referenced to the Au 4f_7/2_ at 84.0 eV [105].

### 5.4 Near‐Edge X‐ray Absorption Fine Structure Spectroscopy

The NEXAFS spectra were collected at the same beamline as the XPS data. The spectra were recorded at the C K‐edge in the partial electron yield mode with a retarding voltage of − 150 V. The incidence angle of the primary X‐rays was varied. The energy resolution was ∼ 0.3 eV. The photon energy scale was referenced to the pronounced π* resonance of HOPG at 285.38 eV [106].

### 5.5 Circular Dichroism

Thin films were measured on SAM/Ni(30 nm)/Sapphire using a JASCO J‐1500 CD spectrophotometer (JASCO Corporation, Tokyo, Japan). Results were collected in the range 205 – 500 nm with 200 nm∙min^−1^ scan speed, data pitch of 0.1 nm, digital integration time of 2 s, and a 1 nm bandwidth. Measurements of BNP solutions (0.01 mM in ethanol) were done in a quartz cuvette (path length 1 cm) over the same wavelength with 1000 nm∙min^−1^ scan speed, data pitch of 0.5 nm, digital integration time of 0.125 s, and a 1 nm bandwidth.

### 5.6 X‐ray Reflectivity

The SmartLab diffractometer (Rigaku Corporation, Tokyo, Japan) was used with monochromatic Cu Kα_1_ radiation, with a divergent slit with parallel‐beam optics and a 2 mm Soller slit. Fit was done using the GenX software.

## Author Contributions

The concept of the study was developed jointly by A.N., C.P., M.T., and P.K. They also prepared the manuscript. A.N. and C.P. fabricated the samples and carried out AFM, XRR, SQUID measurements. A.G. and R.N. provided the mc‐AFM measurements. M.Z. conducted XPS and NEXAFS experiments and provided the interpretation of the results. F.S. and G.S. carried out CD experiments. A.N., C.P., A.G., M.Z., and F.S. contributed to the data analysis.

## Conflicts of Interest

The authors declare no conflicts of interest.

## Supporting information




**Supporting File**: advs76252‐sup‐0001‐SuppMat.docx.

## Data Availability

The data that support the findings of this study are available from the corresponding author upon reasonable request.
