# Supplementary material for: Chiral‐Induced Spin Selectivity Effect in a 1 nm Thin 1,1′‐Binaphthyl‐2,2′‐diyl Hydrogenphosphate Self‐Assembled Monolayer on Nickel Oxide
Source: Adv Sci (Weinh). 2026 Jun 23;13(52):e76252. doi: 10.1002/advs.76252 (PMC13336864; doi:10.1002/advs.76252)
Supplement: Supplementary file 1 [file ADVS-13-e76252-s001.docx]

Supporting Information

Chiral-Induced Spin Selectivity Effect in a 1 nm Thin 1,1’-Binaphthyl-2,2’-diyl Hydrogenphosphate Self-Assembled Monolayer on Nickel Oxide

Abin Nas Nalakath^#^, Christian Pfeiffer^#^, Anu Gupta, Franziska Schölzel, Michael Zharnikov, Georgeta Salvan, Ron Naaman, Marc Tornow*, and Peer Kirsch*

1. Sample Preparation

Samples are based on substrates supplied by SIEGERT WAFER GmbH (Aachen, Germany) and utilize a double-side polished 100 mm highly p-doped (boron, ρ < 0.005 Ωcm) silicon wafer. 10 nm Ti were sputtered on back-and front side as adhesion layers. Subsequently, 100 nm Au and 100 nm Ni were sputtered on the back- and front side, respectively. Native oxides in between deposition steps were removed via ion beam polishing. The 8 × 8 mm^2^ samples were cleaned via acetone and isopropanol sonication. For all (mc-)AFM measurements, the surfaces were oxygen plasma treated at 80 W and 0.3 mbar for 30 s (Smart Plasma, plasma technology GmbH, Herrenberg, Germany) before the self-assembly process. In the case of XPS, XRR, and CD experiments, they were instead treated with UV-ozone (UV Ozone Cleaner UVC-1014, NanoBioAnalytics, optical power 4 W and 254 nm) for 15 minutes. Samples were immersed in a 10 mM solution of 1,1’-binaphthyl-2,2’-diyl hydrogenphosphate (BNP) solution in tetrahydrofuran (THF) or ethanol for 72 h. *R-, S-,* and *rac*-BNP were obtained from Sigma-Aldrich. After immersion, samples were annealed at 80 °C for 1 h, rinsed with isopropanol, and again annealed at 80 °C for 10 min. SAM-coated samples were fabricated for both chiral enantiomers (*R-*BNP and *S-*BNP) and a racemic mixture of both enantiomers (*rac-*BNP).

2. X-ray Photoelectron Spectroscopy (XPS)

The measurements were carried out at the bending magnet HE-SGM beamline of the synchrotron storage ring BESSY II in Berlin, Germany. This beamline provides a linearly polarized X-ray light with a polarization factor of ~90 %. A custom-designed experimental station was used.[1] All measurements were performed at room temperature. The experiments were conducted in ultra-high vacuum, at a base pressure of ca. 1×10^–9^ mbar. The XPS spectra were measured with a Scienta R3000 electron energy analyzer, in normal emission geometry. The primary photon energy (PE) was set to 350 or 580 eV to access specific core levels and to vary the surface sensitivity. The energy resolution at these PEs was ~0.3 eV and ~0.5 eV, respectively. The binding energy (BE) scale of the spectra was referenced to the Au 4f_7/2_ emission of the Au substrate at 84.0 eV.[2] For the evaluation of the XPS data, we used the standard expressions for the attenuation and self-attenuation of the photoemission signals from the substrate and an overlayer[3] and the literature values for the attenuation lengths of these signals in SAM-like films.[4] The data for the binol SAMs were referenced to those for *n-*hexadecanethiolate (C16) monolayer on Au(111). This particular system serves as an established reference for SAM studies and is characterized by a thickness of 1.89 nm and a packing density of $4.63\cdot{10}^{14}$ molecules$\cdot$cm^-2^.[5, 6]

The thickness of the substrate oxide film, $d_{\mathrm{NiO}}$, was estimated based on the relative weights of the oxide and metal contributions in the Ni 3p spectra of the samples (see Figure S1 as an example).

$$\begin{aligned} d_{\mathrm{NiO}}=\lambda\ln[(I_{\mathrm{NiO}}+I_{\mathrm{Ni}})/I_{\mathrm{Ni}}],\#\left( 1 \right) \end{aligned}$$

where $\lambda$ is the attenuation length of the Ni 3p signal at the given kinetic energy (1.1 nm)[4] and $I_{\mathrm{NiO}}$ and $I_{\mathrm{Ni}}$ are the relative intensities of the Ni oxide and Ni metal signals, respectively. The derived $d_{\mathrm{NiO}}$ values varied from 1.07 to 1.47 nm for the different samples, with the average value of 1.3 nm.

The thicknesses of the BNP and C14 SAMs on Ni (hydrocarbon matrix only) were calculated based on the intensity of the C 1s signal for these films, referenced to the value for C16/Au. The packing densities of the BNP SAMs were calculated based on the intensities of the P 2p signals, referenced to the value for C14/Ni. The different attenuation of the P 2p signal by the binol and alkyl matrices was accounted for. It was assumed that the packing density of C14/Ni is similar to that of C16/Au.

**Figure S1:** As measured Ni 3p XP spectrum of *R*-BNP on Ni, representative of the entire sample series. The spectrum is decomposed in the contributions associated with metal Ni and NiO_x_.

3. Near-edge X-ray Absorption Fine Structure Spectroscopy (NEXAFS)

The NEXAFS spectra were collected at the carbon K-edge in the partial electron yield mode with a retarding voltage of $-$150 V. The incidence angle of the primary X-rays was varied between the normal (90°; the **E** vector parallel to the sample surface) and grazing (20°; the **E** vector nearly perpendicular to the sample surface) incidence geometry to monitor the linear dichroism reflecting the molecular orientation in the SAMs.[7] The energy resolution was ~0.3 eV. The photon energy (PE) scale was referenced to the pronounced π* resonance of HOPG at 285.38 eV.[8] The raw spectra were corrected for the PE dependence of the incident photon flux and reduced to the standard form with zero intensity in the pre-edge region and the unity jump in the far post-edge region.

Representative C K-edge NEXAFS spectra of *S-*BNP, *rac-*BNP, and C14 on Ni are presented in Figure S2. The spectrum of the reference C14/Ni in Fig. S2c shows the characteristic resonances of the alkyl chains[9–11], meaning C-H band at ~287.7 eV, comprised of several σ*C-H / Rydberg resonances, and σ* C-C and σ* C-C´ resonances at ~293.4 eV and ~302 eV, respectively. These resonances exhibit a pronounced linear dichroism characteristic of well-ordered alkyl chains in upright orientation.[11] Specifically, the C-H band, with the transition dipole moment (TDM) perpendicular to the alkyl backbone[12], has a higher intensity at **E** parallel to the sample surface (90°). The extent of the linear dichroism in the spectra of C14/Ni (Figure S2c) is similar to that for C16/Au (Figure S3), suggesting a similar molecular orientation with an average tilt angle of 30-35°, and supports the assumption about a similar packing density in these SAMs. A further important implication is the possibility of preparing SAMs of comparable quality by the phosphoric acid anchoring on oxidized Ni, as by thiolate anchoring on Au(111). Analogous quality can therefore be expected for BNP, which is indeed supported by the spectra *S-*BNP/Ni and *rac-*BNP/Ni in Figures S2a and S2b, respectively, showing the characteristic absorption features of naphthalene[13–15] – the major building block of BNP. It is above all the double π_1a_* and π_1b_* resonances at ~284.75 eV and ~285.7 eV, with the characteristic intensity relation between both peaks, followed by weaker and less pronounced π* and σ* resonances at ~288.5 eV, ~290.2 eV, ~293.8 eV, and ~301 eV. Significantly, the difference spectrum of *S-*BNP/Ni exhibits a small but pronounced linear dichroism at the positions of the π_1a_* and π_1b_* resonances, which indicates a certain degree of orientational order in this monolayer. Considering that the respective TDMs are oriented perpendicular to the plane of the naphthalene moieties, higher intensities of these resonances at **E** parallel to the sample surface (90°) suggest a predominant upright orientation in these films. A qualitative evaluation of the NEXAFS data following the formalism for a vector-like orbital[7, 11] gave an average tilt angle of the naphthalene moieties in the backbone of the *S-*BNP/Ni at $\sim$31 ± 3 °. In contrast, there is no dichroism in the spectra of the *rac-*BNP, indicating that this film is most likely disordered and of lower quality than the enantiomer monolayers, in full agreement with the XPS data.

**Figure S2:** C K-edge NEXAFS spectra of *S-*BNP (a), *rac-*BNP (b), and C14 (c) on Ni (the spectra of *R*-BNP are very similar to that of *S*-BNP). The data include the spectra acquired at the different X-ray incidence angles, marked at the spectra (black lines), and the difference spectra resulting from subtracting the spectrum acquired at an X-ray incidence angle of 90° from that acquired at 20° (blue lines). The most prominent resonances are assigned. Horizontal, black dashed lines correspond to zero.

**Figure S3:** C K-edge NEXAFS spectra of C16/Au. The data include the spectra acquired at the different X-ray incidence angles, marked at the spectra (black lines), and the difference spectra resulting from subtracting the spectrum acquired at an X-ray incidence angle of 90° from that acquired at 20° (blue lines). The most prominent resonances are assigned. Horizontal, black dashed lines correspond to zero.

4. Atomic Force Microscopy

To characterize the surface morphology of the BNP SAMs on nickel, AFM in both tapping and contact mode was used. More specifically, the Dimension V AFM (Bruker/Veeco, Billerica, Massachusetts, United States) was utilized with the diamond-like-carbon-coated tips 190DLC (tip radius: 15 nm) from BudgetSensors (Sofia, Bulgaria). The instrument was controlled, and the images were recorded using the Nanoscope 7 software. In tapping mode, 5 × 5 µm^2^ images were taken. To determine the SAM thickness, the so-called “scratching” method was applied on a 1 × 1 µm^2^ area.[16] The voltage setpoint that applied the force to achieve this was calibrated so that the bare nickel substrate below was not damaged. Subsequently, a 3 × 3 µm^2^ tapping image was taken. The height difference between the “scratched” and “unscratched” areas of the image indicates the SAM thickness. The AFM data was analyzed using Nanoscope and Origin.

Tapping measurements of both bare and SAM-coated samples are shown in Figure S4. The bare Ni surfaces (Fig. S4a) exhibit an average root-mean-square (RMS) roughness of $r_{\mathrm{RMS}}=0.6 \pm0.1 \mathrm{nm}$. For the three SAM-coated samples (*R-*BNP in Fig. S4b, *S-*BNP in Fig. S4c, and *rac-*BNP in Fig. S4d), some agglomerations are visible, indicating that the molecules did not form a perfect monolayer in these regions. Because of these spots, the average RMS roughness increases (e.g., $r_{\mathrm{RMS}}$ > 1 nm for the *R-*BNP sample in Fig. S4b). However, in the areas in between these presumed molecule agglomerations, the roughness is in the same range as for the bare Ni surface (Fig. S4a). Therefore, no difference in roughness can be observed for bare Ni surfaces compared to surface areas with expected SAM. Given the low molecular length of BNP ($\approx$1 nm), this finding aligns with previous publications and indicates the presence of well-ordered SAMs: Typically for phosphonic acids, the roughness of TiN substrates was preserved after the deposition of a roughly 0.8 nm thick SAM of 6-aminohexylphosphonic acid. Further, for Al substrates of much higher roughnesses (> 30 nm), no significant change in roughness was reported after the growth of alkyl phosphonic acid monolayers.[17] Similarly, for thiol-based SAMs, it was shown that neither the much longer alkane chain 16-mercaptohexadecanoic acid nor the thiolated biphenyl derivative 1, 1’-biphenyl-4-thiol (BPT) altered the roughness of an Au substrate.[18] A monolayer of the cross-linked version of BPT even led to a decrease in roughness.[18, 19]

**Figure S4:** Representative tapping mode images for the bare Ni substrate (a) and Ni substrates coated with *R-*BNP (b), *S-*BNP (c), and the racemic mixture *rac-*BNP (d).

The results of the “scratching” experiments are presented in Figure S5. The recordings Fig. S5a and Fig. S5b depict the tapping-mode images after scratching *R-*BNP and *rac-*BNP-coated samples, respectively. As is visible, a central square of lower height is apparent for all three SAMs (see Fig. 3d, main paper), indicating successful mechanical removal of the soft organic layer. Height variations along the horizontal axes of the images are averaged over the scratched areas and plotted in Fig. S5c and Fig. S5d for *R-*BNP and *rac-*BNP, respectively. Here, areas with agglomerations left in the supposedly scratched areas are omitted.

The resulting SAM thickness determinations – as calculated via the height differences between the scratched and unscratched parts in the profiles – are presented in Table S1. The height peaks to the left and right of the scratched center are caused by the accumulation of molecules being pushed aside by the AFM tip and are, therefore, not considered for the calculation. As can be seen, all calculations via the height profile yield a layer thickness ≥ 0.5 nm. The scratching experiment for the *R-*BNP samples (Fig. S5d) results in a thickness of $\sim$ 0.8 nm, which matches closest with the theoretical molecule length of 0.96 nm.

**Figure S5:** AFM scratching experiments. Upper row: (a) and (b) depict the 3 × 3 µm^2^ tapping recordings of scratched samples of *R-*BNP and *rac-*BNP, respectively. Lower row: mean height profiles along the horizontal axis over the scratched areas in (a) and (b) are depicted in (c) (*R-*BNP) and (d) (*rac-*BNP), respectively. Here, horizontal profiles that include molecule agglomerations in the central scratched area (as in the center in (b)) were omitted.

**Table S1:** SAM thickness estimated from height profile (Fig. S5c, Fig 3e, and Fig. S5d). Error represents the largest standard deviation of the height profile of the respective scratched or unscratched areas.

| **SAM molecule** | **SAM thickness [nm]**  **determined via height profile** |
| --- | --- |
| *R*-BNP | 0.8 +/- 0.5 |
| *S*-BNP | 0.6 +/- 0.1 |
| *rac*-BNP | 0.5 +/- 0.1 |

**5. X-ray Reflectivity**

The X-ray reflectivity measurements were performed using a SmartLab diffractometer (Rigaku Corporation, Tokyo, Japan), which utilized monochromatic Cu Kα_1_ radiation ($\lambda=1.54056 Å$). The setup includes a divergent slit with parallel-beam optics and a 2 mm Soller slit. Specular $\theta$-$2\theta$ scans were collected over a $2\theta$ range of 0° to 10°, with a step size of 0.01° and a counting time of 0.5 deg·min^-1^. The resulting reflectivity profiles were analyzed and fitted using the GenX software package.

**Table S2:** Estimated thicknesses, roughness, and densities of the layers based on the XRR data.

| **Layer** | **Thickness (nm) (±0.06)** | **Roughness (nm) (±0.01)** | **Density (g/cm^3^)  (±0.532)** |
| --- | --- | --- | --- |
| SiO_2_ | 2.48 | 0.29 | 2.39 |
| Ti | 10.04 | 0.50 | 4.77 |
| Ni | 99.19 | 1.68 | 8.77 |
| NiO | 1.33 | 0.76 | 4.71 |
| BNP | 0.98 | 0.63 | 1.73 |

**Table S3:** Comparison of BNP monolayer thickness values obtained from XRR, AFM scratching, and XPS measurements.

|  | **Theoretical**  (molecule contour length) | **XPS** | **XRR** (*rac-*BNP) | **AFM Scratching**  (*R-*, *S-*, *rac*-BNP) |
| --- | --- | --- | --- | --- |
| BNP SAM thickness (nm) | 0.96 | 0.92 | 0.98 ± 0.09 | 0.4 – 0.8 ± 0.5 |

The surface density of *R-* and *S-*BNP of $\sim1.9\cdot{10}^{14}$ molecules⋅cm^-2^ on NiO/Ni substrates obtained from the XPS measurements agrees very well with the crystal structures:[20, 21] enantiopure *R-* and *S-*BNP crystallize in a layered structure (space group orthorhombic *P* 2_1_2_1_2_1_) with the polar phosphoric acid moieties separated from the aromatic binaphthyl part of the molecules. These layers have orthogonal lattice constants of 0.919 nm and 0.599 nm, indicating a packing density of 1.817 molecules·nm^-2^ within the layers.

In contrast, racemic BNP (space group triclinic *P*-1)[22] does not crystallize in layers like the enantiopure compounds. Therefore, the crystal structure cannot be used as a reference for surface-adsorbed *rac-*BNP. This difference between the structures of enantiopure and racemic BNP might account for the lower experimental packing density of *rac-*BNP as determined via XPS.

**Figure S6:** Top view onto a layer of *S-*BNP (4.6 x 3.0 nm^2^), sliced out of the crystal structure,[21] with a packing density of 1.817 molecules·nm^-2^. A similar structure can be assumed for the monolayer adsorbed to nickel oxide and other substrates.

**6. Circular Dichroism in Solution**

Spectra were recorded with the same instrument as for thin films (see below). The measurements were conducted in solution at room temperature, utilizing a quartz cuvette with a path length of 1 cm and a sample concentration of 0.01 mM in ethanol. Spectra were collected over a wavelength range of 205 to 500 nm, with a scan speed of 1000 nm·min^-1^, a bandwidth of 1 nm, a digital integration time (D.I.T.) of 0.125 seconds, and a data pitch of 0.5 nm. Each spectrum was obtained by averaging five accumulations to enhance the signal-to-noise ratio. The solvent baseline was recorded separately and then subtracted from the sample spectra. Absorbance values were converted to molar extinction coefficients (ε, M^-1^·cm^-1^) using the Beer-Lambert law. CD ellipticity $\left( \theta, \mathrm{mdeg} \right)$ was converted to molar circular dichroism (Δε, M^-1^·cm^-1^) using the relation: $\Delta\varepsilon=\theta/\left( 32980\cdot c\cdot l \right)$, where $c$ in the concentration and $l$ is the path length.

The UV-vis absorption spectra for *R-*BNP, *S-*BNP, and *rac-*BNP are presented in Figure S7 (taken with same instrument as CD). All samples show strong absorption that extends to approximately 350 nm, primarily attributed to intense π-π* transitions of the binaphthyl moiety in the deep UV region (less than 240 nm).[23–25] A strong absorption maximum occurs at 215.5 nm with an absorbance $A$ of approximately 1.20, which corresponds to a molar extinction coefficient $\varepsilon=1.2 \cdot{10}^{5}$mol^-1^⋅cm^-1^. There is also a weaker shoulder at approximately 228 nm, with an absorbance of around 0.90, resulting in an extinction coefficient of $\varepsilon=9.0\cdot{10}^{4}$ mol^-1^⋅cm^-1^. Beyond 320 nm, the spectra decline rapidly, with absorbance values of $A\leq0.10$ and $\varepsilon\leq1.0\cdot{10}^{4}$ mol^-1^⋅cm^-1^. The absorption profiles of the racemic and enantiopure BNP samples are nearly identical, indicating that chirality does not affect the ground-state electronic transitions of BNP.

**Figure S7:** UV absorption spectra of *R-*BNP (red), *S-*BNP (blue), and *rac-*BNP (black) in 0.01 mM ethanol solution

**7. Circular Dichroism (CD) of Thin Films**

For the substrate, 30 nm Ni was sputtered onto sapphire samples (C-cut(001), 0.5 mm, double side polished, supplied by CrysTec) using a home-built radio frequency sputtering system. Ar was used as the working gas. BNP was deposited on the Ni films using the above-described SAM deposition procedure. CD thin film measurements were performed using a JASCO J-1500 CD spectrophotometer (JASCO Corporation, Tokyo, Japan). Spectra were collected in the wavelength range of 205-500 nm with a scan speed of 200 nm·min^-1^, a data pitch of 0.1 nm, a digital integration time of 2 s, and a bandwidth of 1 nm. Samples were mounted on a custom-built rotation stage that enabled automated rotation in 15° steps between azimuthal angles of 0° and 180°. Measurements were acquired for both front and back configurations. To extract the intrinsic CD response, spectra are averaged over all rotation angles as well as the front and back measurements. Furthermore, the spectrum of a bare Ni reference sample was subtracted, isolating the contribution of the BNP layer.

For comparison, the UV-Vis and CD spectra for *S*-BNP were also calculated by time-dependent density functional theory (TD-DFT) on the TD-CAM/6-311+G(d,p) level of theory.[26] The calculations provided the same signs for the respective CD signals, thus confirming the absolute configuration of the compound.

8. Magnetic Conductive Atomic Force Microscopy

8.1. Method Description

The chiral-induced spin selectivity effect was measured via its induced magnetoresistance (CISS-MR) using a custom-built mc-AFM setup. The system includes a Beetle Ambient AFM and an electromagnet controlled by an R9 electronic controller (RHK Technology, Troy, MI, USA). Pt-coated conductive tips of the type DPE-XSC11 (MikroMasch, Sofia, Bulgaria) with a spring constant of 3 - 5.6 N m^-1^ and a tip radius < 40 nm were utilized. The presented current-voltage (I-V) characteristics are based on 40-62 I-V sweeps for both magnetic field directions, while the tip was kept over the same position. During the measurements, a magnetic field of ~ 0.5 T was applied upwards or downwards perpendicular to the sample plane. The response of the magnetic film to that applied field can be seen in the SQUID measurements (Figure S13 and S14). The applied force to bring the tip in contact with the surface was set to 8-10 nN and kept constant (on a given spot) for both magnetic field directions.

8.2. Statistics

The IV characteristics presented in the main paper are based on five measurement positions for *S*-BNP (individual measurements shown in Figure S8), three measurement positions for *R*-BNP (individual measurements shown in Figure S9), and four positions for *rac*-BNP (individual measurements shown in Figure S10).

**Figure S8:** Individual current-voltage measurements for *S-*BNP-coated sample. Red (blue) lines indicate individual curves taken while the magnetic field was pointing upwards (downwards) perpendicular to the sample plane. Thicker lines indicate the respective mean current for that position. The following number of curves were taken for upwards (downwards) magnetization: Position A: 61 (55), Position B: 55 (54), Position C: 22 (66), Position D: 55 (28), Position E: 60 (63).

**Figure S9:** Individual current-voltage measurements for *R*-BNP-coated sample. Red (blue) lines indicate individual curves taken while the magnetic field was pointing upwards (downwards) perpendicular to the sample plane. Thicker lines indicate the respective mean current for that position. The following number of curves were taken for upwards (downwards) magnetization: Position F: 62 (54), Position G: 50 (54), Position H: 51 (63)

**Figure S10:** Individual current-voltage measurements for *rac*-BNP-coated sample. Red (blue) lines indicate individual curves taken while the magnetic field was pointing upwards (downwards) perpendicular to the sample plane. Thicker lines indicate the respective mean current for that position. The following number of curves were taken for upwards (downwards) magnetization: Position I: 51 (54), Position J: 54 (54), Position K: 40 (51), Position L: 54 (52)

**Figure S11:** Calculated CISS-MR for *R-*BNP (green), *S-*BNP (black), and *rac*-BNP (magenta) over the entire voltage range -1 V to 1 V. A peak outside the shown y-axis is reached between -0.25 V and 0 V for both enantiomers and the racemic mixture. This is a numerical artifact, as both $I_{Mag Up}$ and $I_{Mag Down}$ reach 0 A. This peak not being exactly at 0 V could be a result of the molecule dipole or a slight offset in the mc-AFM measurement setup.

9. Fowler-Nordheim Tunneling Model

For the FN plot, only the data with positive voltages $V$ and currents $I$ are analyzed and transformed to produce a $ln(I/{V^{2}})$ vs $1/V$ plot. To find a linear fit for the FN regime, we follow the relation[27]

$$\begin{aligned} J=\alpha E^{2}\cdot exp\left( -\frac{B}{E} \right)\#\left( 2 \right) \end{aligned}$$

with

$$\begin{aligned} \alpha=\frac{m_{e}}{m^{*}}\frac{q^{3}}{8\pi h\phi_{B}}\#\left( 3 \right) \end{aligned}$$

and

$$\begin{aligned} B=\frac{8\pi}{3}\left( 2\frac{m^{*}}{h^{2}} \right)^{\frac{1}{2}}\frac{\phi_{B}^{\frac{3}{2}}}{q}.\#\left( 4 \right) \end{aligned}$$

Here, $E$ is the electrical field, $J$ the current density, $\phi_{B}$ the effective barrier height, $q$ the electronic charge, $m_{e}$ the electron mass, $m^{*}$ the effective electron mass in the barrier material, and $h$ the Planck constant.[27] Rearranging equation (2) yields

$$\begin{aligned} \ln\left( \frac{I}{V^{2}} \right)=-Bd\cdot\frac{1}{V}+\ln\left( \frac{S\alpha}{d^{2}} \right).\#\left( 5 \right) \end{aligned}$$

For relation (5), we set $J=I/S$, where S is the area of the junction, and $E=V/d$, where $V$ is the applied voltage and $d$ is the barrier width (SAM and oxide). Consequently, the FN regime is fitted according to the linear equation $y=s\cdot x+c$, where $y=\ln\left( I/{V^{2}} \right)$, $s=-Bd$, $x=1/V$, and $c=\ln\left( {S\alpha}/{d^{2}} \right)$. In a Python script, an ordinary least squares method is used to find $\theta=\left[ \begin{matrix} m \\ c \end{matrix} \right]$, such that

$$\begin{aligned} \min_{\theta} \left\| A\theta-Y \right\|_{2}^{2},\#\left( 6 \right) \end{aligned}$$

where

$$\begin{aligned} Y=\left[ \begin{matrix} ln({I_{1}}/{V_{1}^{2})} \\ ln({I_{2}}/{V_{2}^{2})} \\ \begin{matrix} \vdots\\ ln({I_{N}}/{V_{N}^{2})} \end{matrix} \end{matrix} \right], A=\left[ \begin{matrix} 1/{V_{1}} & 1 \\ 1/{V_{2}} & 1 \\ \begin{matrix} \vdots\\ 1/{V_{N}} \end{matrix} & \begin{matrix} \vdots\\ 1 \end{matrix} \end{matrix} \right],\#\left( 7 \right) \end{aligned}$$

and $I_{i}$, $V_{i}$ are the datapoints in the FN regime for current and voltage, respectively.

To get the relative difference between the two effective barriers, we can use

$$\begin{aligned} \frac{\phi_{B\uparrow}}{\phi_{B\downarrow}}=\left( \frac{s_{\uparrow}}{s_{\downarrow}} \right)^{\frac{2}{3}},\#\left( 8 \right) \end{aligned}$$

where $s_{\uparrow}$ and $s_{\downarrow}$ are the slopes of the fitted curves for the cases when the substrate is magnetized upwards and downwards, respectively. As can be seen, the resulting ratio of effective barriers is solely dependent on the two slopes and not on physical parameters. However, to derive the difference in the effective barrier height, a solution needs to be found for

$$\begin{aligned} \left| {\Delta\phi}_{B} \right|=\left| \phi_{B\uparrow}-\phi_{B\downarrow} \right|,\#\left( 9 \right) \end{aligned}$$

where $\phi_{B\uparrow}$ is the effective tunneling barrier for current measured when the ferromagnetic substrate is magnetized upwards and $\phi_{B\downarrow}$ the effective tunneling barrier for current measured when the ferromagnetic substrate is magnetized downwards. In combination with equation (8), this yields

$$\begin{aligned} \left| {\Delta\phi}_{B} \right|=\left| \phi_{B\downarrow}\left[ \left( \frac{s_{\uparrow}}{s_{\downarrow}} \right)^{\frac{2}{3}}-1 \right] \right|,\#\left( 10 \right) \end{aligned}$$

Consequently, to get the effective barrier height difference, the full calculation results in

$$\begin{aligned} \left| {\Delta\phi}_{B} \right|=\left| \left| \frac{3qs_{\downarrow}}{8\pi d} \right|^{\frac{2}{3}}\cdot\left( \frac{h^{2}}{2m^{*}} \right)^{\frac{1}{3}}\cdot\left[ \left| \frac{s_{\uparrow}}{s_{\downarrow}} \right|^{\frac{2}{3}}-1 \right] \right|.\#\left( 11 \right) \end{aligned}$$

For the approximation, the constants were chosen as $q=1.602176634\cdot{10}^{-19} C$, $h=6.62607015\cdot{10}^{-34} J$, and $m_{e}=9.1093837015\cdot{10}^{-31} \mathrm{kg}$. For the remaining parameters, assumptions must be made. The tunneling barrier of the junction consists of two parts: the NiO_x_ ($\sim1.3 \mathrm{nm})$ and the BNP SAM ($\sim1 \mathrm{nm})$. However, we will just model it as a single $d=2.3 \mathrm{nm}$ tunnel barrier with a uniform electric field over the whole junction. In previous literature, the electron mass in alkane SAMs was found to be $0.37m_{e}-0.46m_{e}$ in a Simmons tunneling model.[28] For SiO_2_ tunneling barriers, $0.5m_{e}$ shows to provide the best FN fit for the experimental data.[29] For the fitting in this work, the effective electron mass in the tunneling barrier is chosen to be $m^{*}\approx0.5m_{e}$. The resulting FN plots with the associated fits are shown in Figure S12. For every FN plot, the linear fit was calculated based on the window $\left[ 1V^{-1}, 2V^{-1} \right]$. The derived fit parameters and the relations between $\phi_{B\uparrow}$ and $\phi_{B\downarrow}$ are shown in Table S4.

**Figure S12:** FN plot for the mc-AFM curves for *S-*BNP (a), *R-*BNP (b), and *rac-*BNP (c). Scatter plots indicate data points. Solid lines are the linear fits in the region [1 V^-1^, 2 V^-1^]. Blue color refers to the mean current measured when the substrate was magnetized downwards relative to the sample plane, red color refers to the mean current measured when the substrate was magnetized upwards relative to the sample plane.

**Table S4:** Fitting results for the FN linear fit: slope for linear fit for “Mag Up” (“Mag Down”) current $s_{\uparrow}$ ($s_{\downarrow}$), offset for linear fit for “Mag Up” (“Mag Down”) current $c_{\uparrow}$ ($c_{\downarrow}$), the ratio between the respective effective tunneling barriers (${\phi_{B\uparrow}}/{\phi_{B\downarrow}}$), and the absolute difference between the two effective tunneling barriers $\left| {\Delta\Phi}_{B} \right|$.

|  | $\boldsymbol{s}_{\boldsymbol{\uparrow}}$ **[V]** | $\boldsymbol{c}_{\boldsymbol{\uparrow}}$ | $\boldsymbol{s}_{\boldsymbol{\downarrow}}\boldsymbol{[V]}$ | $\boldsymbol{c}_{\boldsymbol{\downarrow}}$ | ${\boldsymbol{\phi}_{\mathbf{B}\boldsymbol{\uparrow}}}/{\boldsymbol{\phi}_{\mathbf{B}\boldsymbol{\downarrow}}}$ | $\left\vert\boldsymbol{\Delta\phi}_{\mathbf{B}} \right\vert$ |
| --- | --- | --- | --- | --- | --- | --- |
| *R-*BNP | -0.88 | -18.0 | -1.92 | -18.7 | 0.6 | 126 meV |
| *S-*BNP | -1.01 | -19.2 | -0.41 | -18.7 | 1.8 | 91 meV |
| *rac-*BNP | -1.02 | -18.5 | -1.2 | -18.5 | 0.9 | 20 meV |

**10. Superconducting quantum interference device magnetometry**

The magnetic properties of the films were examined using a superconducting quantum interference device (SQUID) magnetometer (MPMS-XL, Quantum Design, San Diego, USA). Measurements were conducted at a temperature of 300 K, with the external magnetic field applied both out-of-plane (OOP) and in-plane (IP) relative to the substrate surface, reaching field strengths of up to 6 T. We subtracted the diamagnetic contributions from the substrate and sample holder from the raw data to ensure accuracy.

Figure S13 displays the magnetic hysteresis loops of a 100 nm Ni thin film, measured in both out-of-plane (OOP, Ni_OP) and in-plane (IP, Ni_IP) orientations. The normalized magnetization $M/{M_{S}}$ is plotted against the applied magnetic field in $\mu_{0}H$. In both orientations, clear ferromagnetic hysteresis loops are observed, with magnetization saturating at approximately $\pm800 \mathrm{mT}$ for the OOP configuration and $\pm400 \mathrm{mT}$ for the IP configuration. This means that with the field applied during mc-AFM measurements, the substrate is not fully saturated, which could lead to an underestimation of the CISS-MR. These values are consistent with previously reported magnetization data for Ni thin films.[30–32] The coercive fields were determined to be 12.2 mT for the OOP orientation and 21.1 mT for the IP orientation, confirming the presence of well-defined ferromagnetic ordering. The distinct differences between the IP and OOP loops highlight the magnetic anisotropy of the film.

**Figure S13:** Magnetization curves of 100 nm Ni thin films measured at 300 K, showing IP (Ni_IP, red) and OOP (Ni_OP, black) magnetization as a function of the applied magnetic field.

To ensure that the magnetic behavior is preserved after the annealing step in the SAM deposition procedure, SQUID magnetization measurements were performed both before and after thermal treatment. The substrate was annealed on a hot plate at 100 °C for 1 hour, after which SQUID measurements were repeated. As shown in Fig. S14, the magnetization curve remains unchanged before and after the heat treatment. The saturation field remains approximately at ± 800 mT.

**Figure S14:** Magnetization as a function of the applied magnetic field of 100 nm Ni thin films measured before heating (black) and after heating at 100 °C for 1 hour (red).

**References**

[1]	A. Nefedov and C. Wöll, *Advanced Applications of NEXAFS Spectroscopy for Functionalized Surfaces, in Surface Science Techniques: Springer Series in Surface Science,* 51st ed. Berlin: Springer-Verlag, 2013.

[2]	J. F. Moulder, W. F. Stickle, P. E. Sobol, and K. D. Bomben, *Handbook of X-ray Photoelectron Spectroscopy*. Eden Prairie, MN, USA: Perkin-Elmer Corporation, 1992.

[3]	J. C. Vickerman and I. S. Gilmore, *Surface analysis: The principal techniques,* 2nd ed. Chichester U.K.: Wiley, 2009.

[4]	C. L. A. Lamont and J. Wilkes, "Attenuation Length of Electrons in Self-Assembled Monolayers of n -Alkanethiols on Gold," *Langmuir*, vol. 15, no. 6, pp. 2037–2042, 1999, doi: 10.1021/la981168p.

[5]	F. Schreiber, "Structure and growth of self-assembling monolayers," *Prog. Surf. Sci.*, vol. 65, no. 5, pp. 151–257, 2000, doi: 10.1016/S0079-6816(00)00024-1.

[6]	M. Zharnikov, "High-resolution X-ray photoelectron spectroscopy in studies of self-assembled organic monolayers," *J. Electron Spectrosc. Relat. Phenom.*, 178-179, pp. 380–393, 2010, doi: 10.1016/j.elspec.2009.05.008.

[7]	J. Stöhr, *NEXAFS Spectroscopy*: Springer-Verlag Berlin Heidelberg, 1992.

[8]	P. E. Batson, "Carbon 1s near-edge-absorption fine structure in graphite," *Phys. Rev. B*, vol. 48, no. 4, p. 2608, 1993.

[9]	S. Shokatian and S. Urquhart, "Near edge X-ray absorption fine structure spectra of linear n-alkanes: Variation with chain length," *J. Electron Spectrosc. Relat. Phenom.*, vol. 236, pp. 18–26, 2019, doi: 10.1016/j.elspec.2019.08.001.

[10]	P. Feulner and M. Zharnikov, "High-resolution X-ray absorption spectroscopy of alkanethiolate self-assembled monolayers on Au(111) and Ag(111)," *J. Electron Spectrosc. Relat. Phenom.*, vol. 248, p. 147057, 2021, doi: 10.1016/j.elspec.2021.147057.

[11]	M. Zharnikov, "Near-edge X-ray absorption fine structure spectroscopy in studies of self-assembled monomolecular films," *J. Electron Spectrosc. Relat. Phenom.*, vol. 264, p. 147322, 2023, doi: 10.1016/j.elspec.2023.147322.

[12]	G. Hähner, M. Kinzler, C. Wöll, M. Grunze, M. K. Scheller, and L. S. Cederbaum, "Near edge x-ray-absorption fine-structure determination of alkyl-chain orientation: Breakdown of the ``building-block'' scheme," *Phys. Rev. Lett.*, vol. 67, no. 7, pp. 851–854, 1991, doi: 10.1103/PhysRevLett.67.851.

[13]	M. B. Robin, I. Ishii, R. McLaren, and A. P. Hitchcock, "Fluorination effects on the inner-shell spectra of unsaturated molecules," *J. Electron Spectrosc. Relat. Phenom.*, vol. 47, pp. 53–92, 1988, doi: 10.1016/0368-2048(88)85005-9.

[14]	Toshihiko Yokoyama, Kazuhiko Seki, Ikuo Morisada, Kunishige Edamatsu, and Toshiaki Ohta, "X-ray absorption spectra of poly-p-phenylenes and polyacenes: localization of π orbitals," *Phys. Scr.*, vol. 41, no. 1, p. 189, 1990, doi: 10.1088/0031-8949/41/1/046.

[15]	H. Ågren, O. Vahtras, and V. Carravetta, "Near-edge core photoabsorption in polyacenes: model molecules for graphite," *Chem. Phys.*, vol. 196, no. 1, pp. 47–58, 1995, doi: 10.1016/0301-0104(95)00091-2.

[16]	A. Szwajca, J. Wei, M. I. Schukfeh, and M. Tornow, "Self-assembled monolayers of alkyl-thiols on InAs: A Kelvin probe force microscopy study," *Surf. Sci.*, vol. 633, pp. 53–59, 2015, doi: 10.1016/j.susc.2014.11.023.

[17]	E. Hoque, J. A. Derose, P. Hoffmann, H. J. Mathieu, B. Bhushan, and M. Cichomski, "Phosphonate self-assembled monolayers on aluminum surfaces," *J. Chem. Phys.*, vol. 124, no. 17, p. 174710, 2006, doi: 10.1063/1.2186311.

[18]	B. Bhushan and H. Liu, "Nanotribological properties and mechanisms of alkylthiol and biphenyl thiol self-assembled monolayers studied by AFM," *Phys. Rev. B*, vol. 63, no. 24, 2001, doi: 10.1103/PhysRevB.63.245412.

[19]	H. Liu, B. Bhushan, W. Eck, and V. Stadler, "Investigation of the adhesion, friction, and wear properties of biphenyl thiol self-assembled monolayers by atomic force microscopy," *J. Vac. Sci. Technol. A.*, vol. 19, no. 4, pp. 1234–1240, 2001, doi: 10.1116/1.1353538.

[20]	J. Bruhn and H. Shah, "CCDC 2260063", 2023.

[21]	I. Fujii and N. Hirayama, "Chiral Space Formed by (+)-(1S)-1,1′-Binaphthalene-2,2′-diyl Phosphate: Recognition of Aliphatic L-α-Amino Acids," *Helv. Chim. Acta*, vol. 85, no. 9, pp. 2946–2960, 2002, doi: 10.1002/1522-2675(200209)85:9<2946::AID-HLCA2946>3.0.CO;2-1.

[22]	S. K. Nayak, S. Chandrasekhar, and T. G. Row, "1, 1′-Binaphthalene-2, 2′-diyl hydrogen phosphate," *Struct. Rep.*, vol. 64, no. 1, o256-o256, 2008.

[23]	D. Amsallem, A. Kumar, R. Naaman, and O. Gidron, "Spin polarization through axially chiral linkers: Length dependence and correlation with the dissymmetry factor," *Chirality*, vol. 35, no. 9, pp. 562–568, 2023, doi: 10.1002/chir.23556.

[24]	F. Mortaheb *et al.,* "Enantiospecific Desorption Triggered by Circularly Polarized Light," *Angew. Chem.*, vol. 131, no. 44, pp. 15832–15836, 2019, doi: 10.1002/ange.201906630.

[25]	T. Kimoto, N. Tajima, M. Fujiki, and Y. Imai, "Control of circularly polarized luminescence by using open- and closed-type binaphthyl derivatives with the same axial chirality," *Chem. Asian J.*, vol. 7, no. 12, pp. 2836–2841, 2012, doi: 10.1002/asia.201200725.

[26]	M. J. Frisch *et al., Gaussian 16 Rev. C.01*. Wallingford, CT, 2016.

[27]	Y. L. Chiou, J. P. Gambino, and M. Mohammad, "Determination of the Fowler–Nordheim tunneling parameters from the Fowler–Nordheim plot," *Solid-State Electron.*, vol. 45, no. 10, pp. 1787–1791, 2001, doi: 10.1016/S0038-1101(01)00190-3.

[28]	W. Wang, T. LEE, and M. A. Reed, "Elastic and Inelastic Electron Tunneling in Alkane Self-Assembled Monolayers," *J. Phys. Chem. B*, vol. 108, no. 48, pp. 18398–18407, 2004, doi: 10.1021/jp048904k.

[29]	Z. A. Weinberg, "On tunneling in metal‐oxide‐silicon structures," *J. Appl. Phys.*, vol. 53, no. 7, pp. 5052–5056, 1982, doi: 10.1063/1.331336.

[30]	C. Nacereddine *et al.,* "Structural, electrical and magnetic properties of evaporated Ni/Cu and Ni/glass thin films," *Mater. Sci. Eng. B*, vol. 136, no. 2, pp. 197–202, 2007, doi: 10.1016/j.mseb.2006.09.021.

[31]	M. Ito *et al.,* "Uniaxial in-plane magnetic anisotropy mechanism in Ni, Fe, and Ni-Fe alloy films deposited on single crystal Y-cut 128° LiNbO3 using magnetron sputtering," *J. Magn. Magn. Mater.*, vol. 564, p. 170177, 2022, doi: 10.1016/j.jmmm.2022.170177.

[32]	K. Santra, Y. Lu, D. H. Waldeck, and R. Naaman, "Spin Selectivity Damage Dependence of Adsorption of dsDNA on Ferromagnets," *J. Phys. Chem. B*, vol. 127, no. 11, pp. 2344–2350, 2023, doi: 10.1021/acs.jpcb.2c08820.
